# Acute effects of a single neurodynamic mobilization session on range of motion and H‐reflex in asymptomatic young subjects: A controlled study

**DOI:** 10.14814/phy2.15748

**Published:** 2023-06-18

**Authors:** Álvaro Cancela, Pablo Arias, Beatriz Rodríguez‐Romero, Marcelo Chouza‐Insua, Javier Cudeiro

**Affiliations:** ^1^ Quirónsalud Hospital A Coruña Spain; ^2^ Neuroscience and Motor Control Group (NEUROcom), University of A Coruña Institute of Biomedical Research of A Coruña A Coruña Spain; ^3^ Department of Physical Therapy, Medicine and Biomedical Sciences University of A Coruña A Coruña Spain

**Keywords:** H‐reflex, muscle stretching exercises, nerve tissue, physical therapy modalities, range of motion articular

## Abstract

Neurodynamic techniques have yielded good clinical results in the treatment of various pathologies. The objective of this study is to examine the short‐term effects of neurodynamic techniques of the sciatic nerve on hip ROM (range of motion) and on the amplitude and latency of the soleus H‐reflex and M‐waves, in young asymptomatic subjects. In a double‐blind controlled trial design, 60 young asymptomatic participants were randomly assigned into six groups with different levels of manipulation of the sciatic nerve. The passive straight leg raise test was used to evaluate the hip ROM amplitude. All evaluations were performed before, 1 min after, and 30 min after intervention. For each time‐point, spinal and muscle excitability were also tested. ROM increased in all groups, but none of the treatment groups had superior effects than the group with no treatment. This means that ROM testing maneuvers increased ROM amplitude, with no add‐on effect of the proposed neurodynamic techniques. Neurophysiological responses changed similarly in all groups, showing that the aftereffects were not intervention‐specific. We observed a significant negative association between the change in limb temperature and the change in latencies of all potentials. ROM‐testing procedures performed repeatedly increase ROM amplitude. This observation should be considered when evaluating the aftereffects of therapeutic interventions on ROM amplitude. None of the explored neurodynamic techniques produced acute aftereffects on hip ROM amplitude, spinal or muscle excitability different to the induced by the ROM testing maneuver.

## Graphical abstract

- One session of neural mobilization does not produce modification of the ROM or the spinal excitability.
- ROM‐testing procedures performed repeatedly increase ROM amplitude.
- There was an association between changes in limb temperature and latencies.

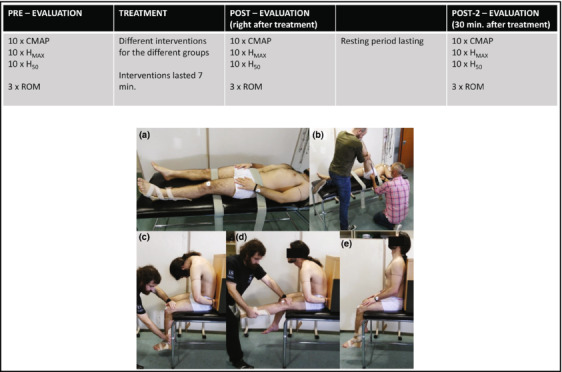

## 1 INTRODUCTION

Neuromeningeal mobilization (also called neurodynamics) (Butler, 1991; Shacklock, 1995)comprises a set of treatment and diagnostic techniques on nerve function. Neurodynamic techniques (NT) include two main techniques: *neural sliding* and *tensile load*. The first involves alternating movements of adjacent joints, which are thought to produce nerve *sliding* (Coppieters et al., 2015), the second is based on a partially sustained elongation of the nerve tract. Both techniques have yielded good clinical results in the treatment of various pathologies and clinical signs, including cubital or carpal tunnel syndrome, non‐radicular back pain, fatigue, spasticity, or improving functional activities in conditions like stroke, nerve palsy, osteoarthritis, fibromyalgia, cervical radiculopathy, or even aging (Anandkumar, 2015; Castilho et al., 2012; De‐la‐Llave‐Rincon et al., 2012; Kim et al., 2016; Nagrale et al., 2012; Oskay et al., 2010; Villafañe, 2013; Villafañe et al., 2011; Villafañe et al., 2012; Villafañe et al., 2013).

However, a note of caution has also been added by Ginanneschi et al. (2015), as neural mobilization in carpal tunnel syndrome may generate conduction failure in peripheral nerves. Moreover, it has been shown that muscle flexibility is associated with the capacity of elongation of neuromeningeal passive structures (McHugh et al., 2012). Some studies indicates that NT increases joint range of motion (ROM) (Castellote‐Caballero et al., 2013; Pietrzak & Vollaard, 2018), which is important in a number of activities with frequent muscle injuries (like sports), as the increase of ROM might protect against muscle injury (Davis et al., 2005). Notwithstanding, it is not unequivocal that sliding and tensile load techniques increases hip and knee ROM (Kaur & Sharma, 2011; Kavlak & Uygur, 2011; Lorentzen et al., 2012; Mafra et al., 2011; Marks et al., 2011; Méndez‐Sánchez et al., 2010; Mhatre et al., 2013; Pagare et al., 2014). Another effect attributed to NT is the modification of spinal excitability, reducing the H‐reflex latency (Kumar & Kaur, 2012; Rezk‐Allah et al., 2011) and increasing its amplitude (Kumar & Kaur, 2012); results not confirmed subsequently in controlled studies (Adel, 2011). Remarkably, many of the above cited studies are case reports or trials with no proper control conditions (Anandkumar, 2015; Castilho et al., 2012; De‐la‐Llave‐Rincon et al., 2012; Kim et al., 2016; Oskay et al., 2010; Villafañe, 2013; Villafañe et al., 2011; Villafañe et al., 2012; Villafañe et al., 2013). Therefore, the objective of this study is to evaluate the aftereffects of NT on ROM amplitude and spinal excitability, under a blind‐controlled design.

## 2 METHODS

### 2.1 Experimental approach to the problem

In a double‐blind controlled design, we tested the effects of a NT single session on hip ROM and H‐reflex excitability in asymptomatic young subjects. Based on previous reports, our hypothesis establishes that NT will increase ROM and H‐reflex amplitude (Castellote‐Caballero et al., 2013; Kumar & Kaur, 2012; Pietrzak & Vollaard, 2018).

The effects NT on H‐reflex and ROM were studied in six groups of 10 subjects receiving different interventions on the sciatic nerve. Subjects were pseudo‐randomly assigned to one of the six study groups, with balanced proportion of men and women, by using EpiInfo software. Subjects were unaware of the existence of different groups and the specific objective of the study.

Investigators (blind to the group assignment) carried out the neurophysiological evaluations and ROM, in this order, at three different time‐points: *pre* and *post* intervention, and again 30 min later (*post‐2*).

Interventions and evaluations were always performed on the subject's dominant limb, defined as: “the leg used to kick a ball” (Kovaleski et al., 1997).

### 2.2 Subjects

Sixty‐seven healthy subjects voluntarily participated in the study. Exclusion criteria were: (i) lower limb surgery in the previous year, (ii) low back pain (iii) positive diagnosis of any neurological disorder.

Sixty subjects (27 women) matched the criteria and were enrolled (mean age, 23.8 years, SD ±2.7; weight 68.0 kg, SD ±10.7; height, 1.7 m, SD ± 0.1).

Subjects received a detailed description of the experimental procedure and the associated risks (potentially low) and signed consent forms. All procedures were approved by our Local Ethics Committee, complying with the Declaration of Helsinki.

### 2.3 Procedures

**FIGURE 1:**
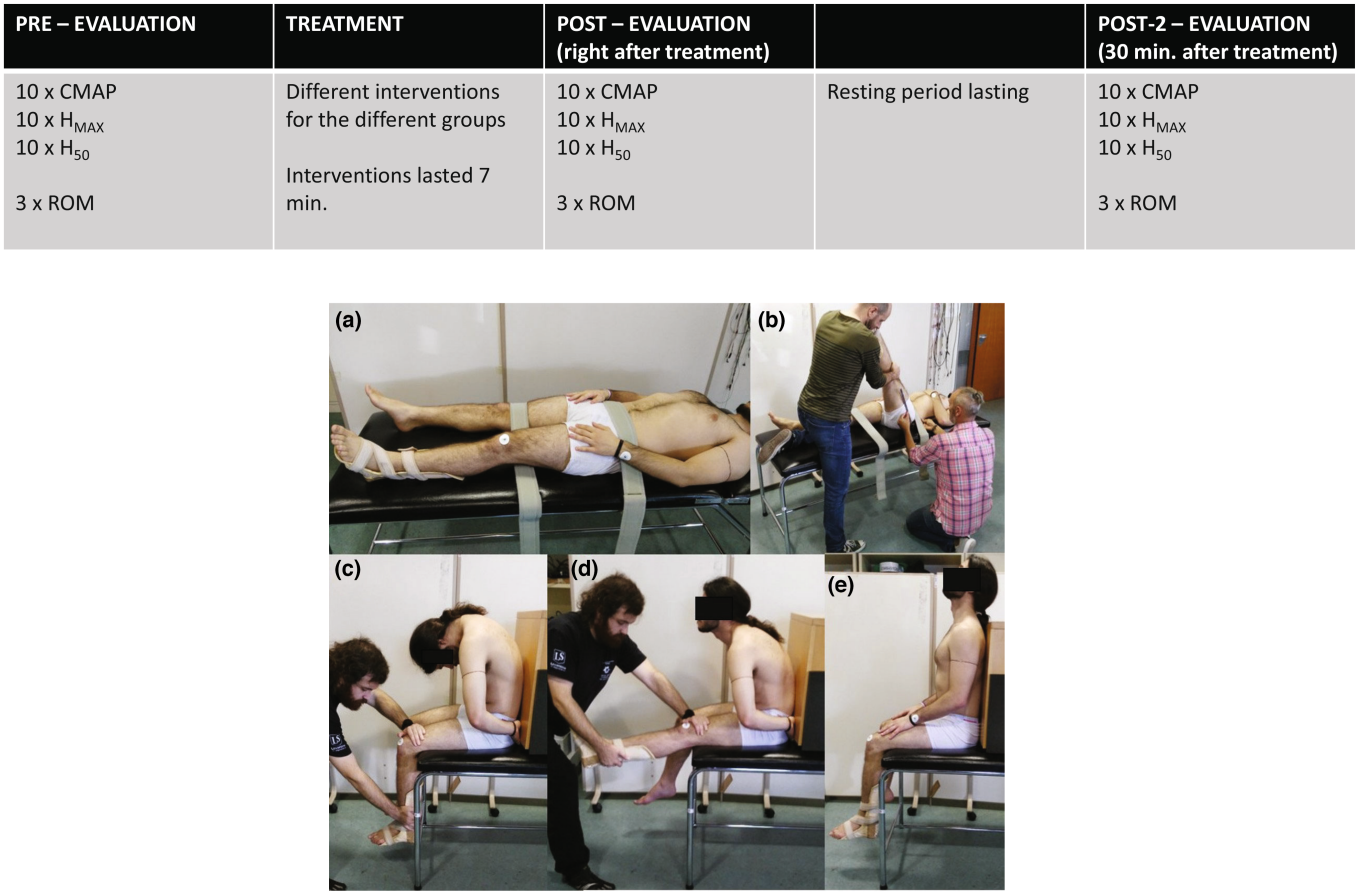
Testing and intervention protocols sequence. (a) Initial position for ROM evaluation. (b) Final position for ROM evaluation. (c) Initial position for neural mobilization group. (d) Final position for neural mobilization group. (e) Position for no‐neural mobilization group.

Figure 1 presents the procedure sequence, including neurophysiological and ROM testing and treatment.

#### 2.3.1 Neurophysiological evaluation

Subject remained seated on a gurney with hips and knees flexed at 90°, with back support. A footstool (adaptable in height) was on the floor to give feet support. Self‐adhesive electrodes were used for recording and applying stimulation.

After skin preparation, the soleus EMG was acquired by means of surface electrodes arranged in a belly–tendon manner, with the reference electrode on the Aquilles tendon and the active electrode on the soleus muscle; the ground electrode was placed ≈4 cm above the active electrode. EMG signals were recorded by means of D360 amplifiers (Digitimer), amplified (×250–1000) and bandwidth filtered between 3 and 3000 Hz. EMG was sampled at 10 kHz and stored in a computer by means of a CED 1401 mkII Power A–D converter (Cambridge Electronic Design), which also triggered a Digitimer DS7A stimulator. Skin temperature was monitored (only in one group, reasons given below) by means of a fine temperature probe attached just at the side of the active EMG recording electrode, the probe was connected to a Cibertec Citer which sampled temperature at 0.1 Hz and delivered the signal to the CED1401; therefore, temperature recording was synchronized to EMG recording.

We stimulated the tibial nerve (1 ms pulses) with the cathode on the popliteal fossa and the anode over the patella. Stimulation pulses were delivered at 0.1 Hz to avoid any induction of post‐activation depression. Different stimulation intensities were used to record the H‐reflex (at two points of the ascending‐leg of the recruitment curve) and the compound muscle action potentials (CMAP). Ten responses were obtained for each intensity.

For each time‐point (pre, post, post‐2), peak to peak amplitude and latency of the following variables were studied (in this order):

1. CMAP acquired at stimulation intensity 20% above the supramaximal.
2. H_MAX_: acquired at the stimulation intensity producing the largest H‐wave amplitude, we also recorded its corresponding M‐wave.
3. H_50_: at *pre* testing, it was acquired at the stimulation intensity inducing H‐wave amplitudes in the ascending leg of the recruitment curve equivalent to the 50% of the H_MAX_. We also recorded its M‐wave. However, for *post* and *post‐2* the stimulation intensity was adjusted to have M‐waves equivalent in amplitude to those obtained at *pre*; this was done to have similar levels of antidromic volleys in the axons of the motoneurons during H‐reflex recording (Goulart et al., 2000; Pinniger et al., 2001).
4. Skin temperature (only recorded in one group) was analyzed in Celsius grades; skin temperature is a valid parameter to estimate muscle temperature as shown previously (de Ruiter et al., 1999).

#### 2.3.2 ROM evaluation

The passive straight leg raise test was used to evaluate the ROM amplitude, this is a valid and reliable test (Ayala et al., 2012; Boyd, 2012). From the previous seated position to record excitability, subjects lain down on the gurney (supine‐position). A strap of velcrum (7 cm‐width) was placed around the gurney and the subject's waist at the level of the anterosuperior iliac spines; another strap secured the nondominant thigh in a similar way; the dominant limb rested on the gurney with knee extension, above the velcrum strap (Position A; Figure 1a). One researcher lifted the dominant leg from the gurney, and then pushed to flex the hip (while keeping knee extension) to the point at which the participant reported the first feeling of muscle tension (Pagare et al., 2014) (this defined Position B), the maneuver lasted ≈3 s. Absence of non‐dominant leg movements was visually checked, continuously. Another examiner performed the goniometric measurement (from position A to B, defined just above) with the goniometer fulcrum placed over the greater trochanter, the fixed‐arm of the goniometer oriented axillary parallel to the gurney, and the moving‐arm aligned to the external femoral condyle (Figure 1b). For each of the three time‐points (*pre*, *post*, *post‐2*), the ROM was tested three times (1 min rest) and the average was calculated.

### 2.4 Treatment

All subjects were seated on a gurney as described above. From this position six different protocols (all of them lasting 7 min; Figure 1) were applied (one per group):

*Neural mobilization (NM)*: This group underwent a slide sciatic nerve maneuver. Subjects adopted the “slump test” position, characterized by trunk flexion with the sacrum in touch with a vertical back‐support Figure 1c, and hips and knees flexed at 90°. A rigid splint was used to maintain a neutral ankle flexion. Participants made an active cervical extension while the knee was passively extended by a researcher to the point of maximum muscle‐tension (identified by each participant), Figure 1d. Then, participants made an active cervical flexion while the researcher performed a passive knee flexion; the whole cycle lasted ≈3 s. It has been advocated that this technique induces a sliding of the sciatic nerve (Ellis et al., 2012). Participants executed the movements during 1 min (Véras et al., 2012); this was a set (i.e., 20 cycles). The whole intervention included four sets, with 1 min of rest between sets.

*No neural mobilization (nNM)*: This protocol was included as a way of controlling the specific maneuvers of the NM group (Lew & Briggs, 1997). The procedure was the same as above, but the participant's whole back was in touch with the vertical back‐support and the neck was kept extended during the 20 cycles (Figure 1e) (Lew & Briggs, 1997).

*Sustained sciatic nerve tension (ST)*: A similar starting position as for NM was used. However, participants maintained cervical flexion and knee extension (to the point of maximum muscle‐tension) for 30 s (without movement), with assistance from the researcher. The intervention included five sets, 1 min rest.

*ROM testing control*: Participants remained at rest for the same period of time as the other groups (7 min). The inclusion of this group controls if ROM testing maneuvers modify ROM amplitude along the protocol. It serves as control for the three groups above presented.

*Control group without ROM testing*: Participants remained at rest as the previous group but without ROM testing. The inclusion of this group controls the effects of ROM testing on spinal excitability.

*Temperature‐control group without ROM testing*: The same as above, but skin temperature was monitored, it serves to control for putative effects of temperature on the neurophysiological tests. This last group was included after performing experiments and data analyses in the other five groups.

### 2.5 Statistical analysis

For each subject, H and M waves amplitudes were normalized with reference to the amplitude of the CMAP at the corresponding evaluation time‐point. The median of the 10 responses acquired at each time‐point was the score introduced in the analyses. CMAP and ROM were considered in absolute values.

First, we tested the assumption of normality of distributions for each variable and group (KS for one sample test).Then, results at the three testing points for M‐wave amplitude of the H_50_ was evaluated with a repeated measures analysis of variance (3×6 ANOVA_RM_) with factor TIME (*pre‐post‐post2*) and GROUP (six groups). Note that the intensity of stimulation for acquiring the M‐wave of the H_50_ was adjusted at *post* and *post‐2* to match the amplitude of the responses obtained at *pre* (see above). For this reason this variable was analyzed in isolation. The same approach was used to evaluate the M‐wave of the H_MAX_, CMAP, and ROM.

For the amplitudes of the two H‐waves, the same statistical model was used but adding factor RECRUITMENT. This way, the analysis allowed us to estimate if the effects were differently expressed for H_50_ and H_MAX_; therefore, if factor RECRUITMENT interacted significantly with any other factor, the estimated effect would be different for H_50_ and H_MAX._ We proceeded the same way to analyze all wave‐latencies, but in the case of M‐waves latencies RECRUITMENT had three levels (M‐wave for H_50;_ M‐wave for H_MAX_, CMAP).

For the ANOVA, the degrees of freedom were corrected with Greenhouse–Geisser coefficients if the sphericity was not assumed.

With data derived from the *temperature‐control group without ROM testing*, we performed correlation analyses to look for the relation between putative changes in H and M waves scores (in latency and amplitude) with changes in temperature; for this purpose we used Pearson's or Sperman's correlation analyses, depending on the violation of normality of the variables.

Significance was set at a *p* < 0.05. Data in the graphs are means and standard deviation (SD).

## 3 RESULTS

### 3.1 Effect of interventions on the M‐wave amplitude of the H_50_

**FIGURE 2:**
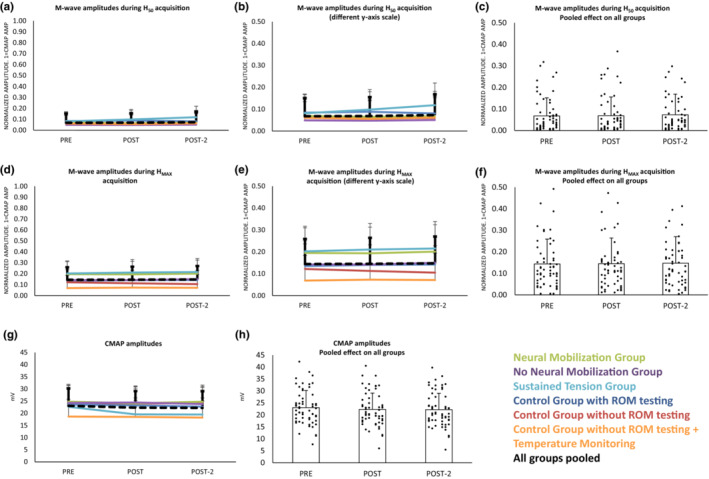
(a) Amplitudes of the M‐waves during the recording of the H_50_ along the three time‐points, in the six groups. Amplitudes were unchanged the along testing times in all groups. (b) The same plot as (a) at different *y*‐axis scale. (c) The same variable that (a) and (b) but pooling groups as their responses along time were not different for the groups, dots are individuals' responses. (d) M‐wave amplitudes during H_MAX_ recording at the testing time‐points, for all groups. (e) The same variable that (d) at different *y*‐axis scale. (f) The same variable that (d) and (e) but pooling groups, since their responses along time were not different for the groups, dots are individuals' responses. (g) CMAP amplitudes at the testing time‐points, for the six groups. (h) The same variable that (g) but pooling groups since their responses along time were not different, dots are individuals' responses. Amplitudes remained unchanged in all groups along time. Scores are means and standard deviations.

M‐wave amplitude during H_50_ acquisition was stable along the three testing time‐points (*F*_2,108_ = 0.8_ε = 0.6_ *p* = 0.4_TIME_) in the six groups (*F*_10,108_ = 1.2_ε = 0.6_ *p* = 0.3_TIME × GROUP_); this was expected as it was imposed by the experimental condition to control the level of antidromic volleys in the axons at the different testing times during H_50_ acquisition. Figure 2a,b show the results split by groups, and Figure 2c with all groups pooled (as the effect along time was not different for each group).

### 3.2 Effect of interventions on the M‐wave amplitude of the H_MAX_

The size of the M‐wave (acquired with the optimum intensity to get the H_MAX_) did not change along the three testing points (*F*_2,108_ = 0.6 *p* = 0.9_TIME_) and this was not different for the six groups (*F*_10,108_ = 0.1 *p* = 1.0_TIME × GROUP_) (Figure 2d–f)_._

### 3.3 Effect of interventions on the CMAP amplitude

The amplitude of the CMAP changed along the three testing points (*F*_2,108_ = 4.2_Ԑ = 0.6_ *p* = 0.039_TIME_). No significant interactions by group was detected indicating that the effect was not different in the six groups. The effect was small and *post hoc* analyses between pairs did not show significant differences between any pair (*Bonferroni p* > 0.1 in all cases) (Figure 2g,h).

### 3.4 Effect of interventions on the H‐waves amplitudes

Contrary to the case of the M‐waves (in which *M‐wave amplitude of the H*_*50*_ had been matched in *post* and *post‐2* to *pre* values), the H‐waves (H_50_ and H_MAX_) were introduced in the same ANOVA to know if the protocol had a different impact on both waves.

**FIGURE 3:**
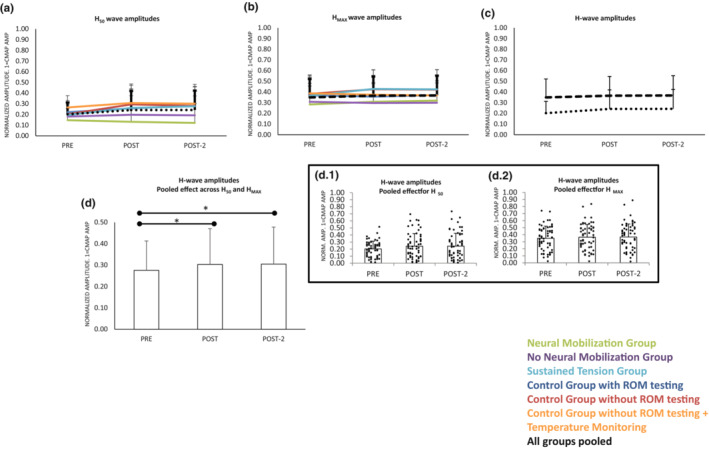
Amplitudes of the H_50_‐waves (a), and the H_MAX_ (b) along the three time‐points in the six groups, responses between groups along pre‐post‐post2 did not differ significantly. (c) Same plot that (a) and (b) but pooling groups, responses for H_50_ did not differ in a significant way from responses for H_MAX_, both increased along time as shown in (d). Insets d.1 and d.2 represent individuals' responses. Scores are means and standard deviations. *p < 0.05.

Two main effects were observed. First, H_MAX_ amplitudes were larger than H_50_ (*F*_1,54_ = 106.2 *p* < 0.001_RECRUITMENT_). Second, amplitudes were modified along the three testing points (*F*_2,108_ = 6.1_ε = 0.9_ *p* = 0.005_TIME_). No significant interactions between them or with factor Group were detected, which means that the effect was not different for all groups (including the Control groups). Subsequent *post hoc* analyses indicated an increase in the amplitudes (H_MAX_ and H_50_) from *pre* to *post (Bonferroni p* = 0.015), which remained at *post‐2 (Bonferroni p* = 0.028). The mean increase from *pre* to *post* was small in size (<3% in the two cases, normalized in terms of their respective CMAP; Figure 3a–d).

### 3.5 Effect of interventions on the M‐waves latencies

**FIGURE 4:**
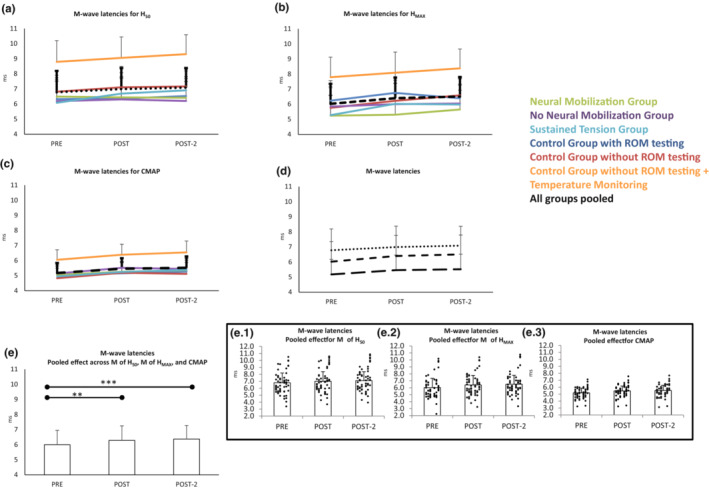
M‐wave latencies changes along time for the different groups when acquiring H_50_ (a), H_MAX_ (b), and CMAP (c). Section (d) shows the same responses with groups pooled (as responses along time did not differ for the groups). Responses for the three types of M responses along pre‐post‐post2 did not differ between them, the increase was significant as shown in section e. Insets e.1, e.2 and e.3 represent individuals' responses. Scores are means and standard deviations. **p < 0.01; ***p < 0.001.

The ANOVA including all M‐waves indicated that the latencies were different for the three recruitment points (*F*_2,108_ = 58.2 *p* < 0.001_RECRUITMENT_). As expected, *post hoc* analyses showed that latency was shorter for the M_max_ (i.e., CMAP) compared to M‐wave of H_50_ and M‐wave of H_MAX_ (*Bonferroni p* < 0.001 for all comparisons). Wave latencies were significantly delayed (≈0.4 ms) along the three testing points (*F*_2,108_ = 16.2_ε = 0.7_ *p* < 0.001_TIME_; *pre* vs. *post Bonferroni p* < 0.01, *pre* vs. *post‐2 Bonferroni p* < 0.001). The lack of significant interactions of factor TIME with any other factor indicated that latencies increased not differently in all M waves explored, along the three testing times and in all groups (Figure 4a–e).

### 3.6 Effect of interventions on the H‐waves latencies

**FIGURE 5:**
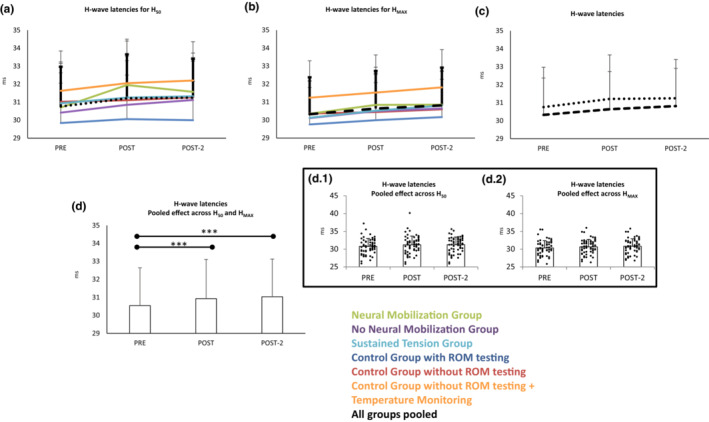
H‐wave latencies changes along time for the different groups when acquiring H_50_ (a) and H_MAX_ (b). Section (c) shows the same responses with groups pooled (since responses along time did not differ in a significant level for the groups). Responses for the two types of H responses along pre‐post‐post2 did not differ between them, both increased as shown in section (d). Insets d.1 and d.2 represent individuals' responses. Scores are means and standard deviations. ***p < 0.001.

The latency of the H‐waves was different for the two recruitment points tested_,_ being H_MAX_ shorter than H_50,_ (*F*_1,58_ = 27.2 *p* < 0.001_RECRUITMENT_; see Figure 5a–c). They were significantly delayed along the three testing points (*F*_2,108_ = 21.8_ε  = 0.9_ *p* < 0.001_TIME_; *pre* vs. *post Bonferroni p* < 0.001; *pre* vs. *post*‐2 *Bonferroni p* < 0.001 – Figure 5d); changes were ≈0.5 ms in magnitude. No significant interactions indicated that the significant increase in latency was not different for H_MAX_ and H_50_ (i.e., both were equally modified), the effects were observed in all groups.

### 3.7 Effect of interventions on the ROM

The ROM was tested in four out of the six groups. Three groups underwent intervention techniques (NM, nNM and ST) and a fourth group only executed the ROM testing technique; the other two groups, without ROM testing, served to control the putative effect of ROM testing and superficial leg temperature on excitability scores.

**FIGURE 6:**
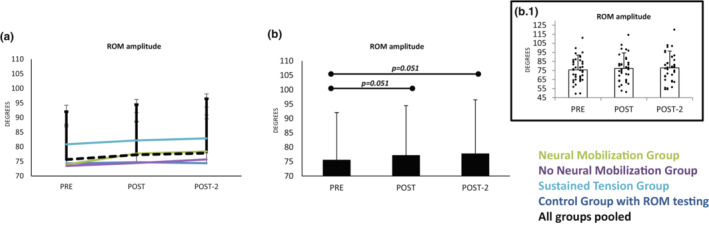
(a) Hip ROM amplitude change along the three testing times for the different groups. Section (b) shows the same responses with groups pooled (as responses along time did not differ between groups). In all groups, ROM increased from PRE to POST, and the change remained at post2. Inset b.1 represents individuals' responses. Scores are means and standard deviations.

The analyses indicated an increase of ROM along the three testing points (*F*_2,72_ = 4.4_ε  = 0.8_ *p* = 0.015_TIME._). The increased was not different in the four groups evaluated (*F*_6,72_ = 0.9_ε  = 0.8_ *p* = 0.470_TIME × GROUP._). Post hoc analyses indicated an increment from *pre* to *post (Bonferroni* p = 0.051) which remained at post‐2 (vs. *pre*; *Bonferroni p* = 0.051); the size of the effect was small, about two degrees. Therefore, the testing protocol itself increases the ROM and NM, nNM and ST interventions had no effect on increasing ROM beyond the explained by ROM testing itself (Figure 6); however, the change is very small.

### 3.8 Effect of limb temperature change on outcomes

Thus far, the results indicated that the effects observed on M and H waves amplitudes and latencies where not different in the six groups tested in our experiment. A same pattern had been observed initially before including in the analyses of the *temperature‐control group without ROM testing*. As the inclusion of this group did not change the behavior of the amplitudes and latencies of the different potentials, we performed correlation analyses between the changes in amplitudes (and latencies) from pre to post and pre to post‐2 versus changes in skin/muscle temperature (pre to post and pre to post‐2). Analyses were performed pooling M‐waves and H‐waves together. These analyses were aimed at understanding the putative effect of limb temperature change on H and M waves parameters.

**FIGURE 7:**
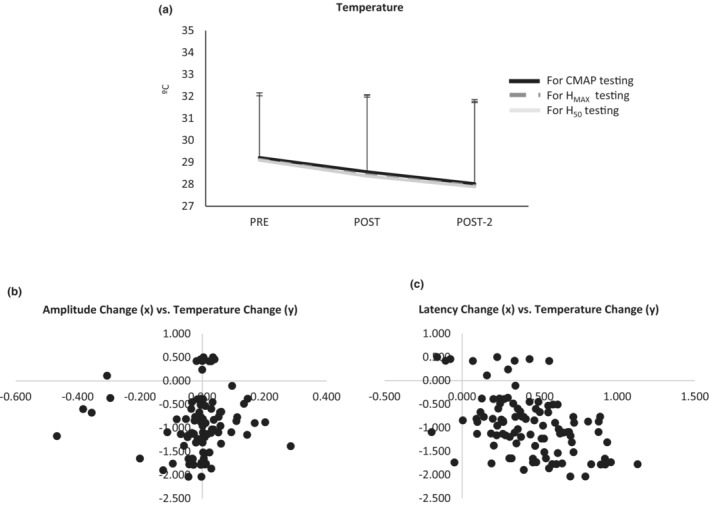
(a) Change in limb temperature during the testing of the potentials at the different time‐points (means and standard deviations). (b) Scatter‐plot of the association between the changes the amplitude of the potentials along the whole protocol (normalized scores – *x* axis) and changes in limb temperature (° – *y* axis). (c) Scatter‐plot of the association between the changes the latency of the potentials along the whole protocol (ms – *x* axis) and changes in limb temperature (° – *y* axis). Changes in latencies were significantly associated to changes in limb temperature; this effect was not observed for the amplitudes of the potentials.

The limb temperature drop along the protocol (keeping the same conditions as in the other groups). This effect was observed within each testing‐time‐point, which means there is a drop in temperature during sequential recording of CMAP, H_MAX_ (an its corresponding M‐wave) and H_50_ (an its corresponding M‐wave); the effect was very small but consistently expressed in the subjects *F*_2,18_ = 17.7 *p* < 0.001_POTENTIAL‐TYPE_. The same was observed along the three testing time‐points *F*_2,18_ = 29.6_ε  = 0.5_ *p* < 0.001_TIME_. There was not interaction between these two factors (see Figure 7a).

The association between the changes in the amplitude of the potentials and the changes in limb temperature along the testing time‐points was not significant (*p* = 0.147, Spearman Rho, Figure 7b). Conversely, the changes in limb temperature and the latencies of the potentials were significantly associated (*p* < 0.001, Pearson), with a negative coefficient (*r* = −0.46), thus a drop in temperature caused latency to increase, Figure 7c.

## 4 DISCUSSION

NT has been advocated to induce changes in ROM and spinal excitability, which made them appealing for clinical practice (Castellote‐Caballero et al., 2013; Kumar & Kaur, 2012; Pietrzak & Vollaard, 2018; Rezk‐Allah et al., 2011). Since it is recognized that some of these studies are not properly controlled (Kumar & Kaur, 2012; Pietrzak & Vollaard, 2018), our objective was to investigate the effect of different NT on ROM and spinal excitability, under better controlled conditions. We have shown that none of the techniques evaluated presents any significant effect on ROM amplitude after a single session, apart from the induced by ROM testing maneuvers itself. None of the protocols tested produced changes in the spinal excitability other than the small (but significant) changes observed in passive control groups, suggesting that the evaluated techniques are not useful to modify spinal excitability, if applied in a single session.

### 4.1 Effect of the protocol on ROM

The ROM increased in all groups which underwent ROM testing. The fact that none of the interventions was able of inducing ROM increments beyond the observed in the ROM testing control group is truly relevant. The absence of such control group in a number of previous studies (Kaur & Sharma, 2011; Méndez‐Sánchez et al., 2010) conditions the interpretation of their results. This is not the case, however, of the study by Castellote‐Caballero et al (Castellote‐Caballero et al., 2013), where a proper control was included and the effectiveness of NT was shown. It is noteworthy, that their study included participants with already limited hip ROM (as inclusion criteria); thus, their participants had a mean ROM before treatment of 58° (with a cut‐off stablished at 75°), whereas our sample of subjects had mean ROM at *pre* larger than 75°.

Some other substantial aspects can explain the differences between the two works. First, the presence of a ceiling effect for ROM amplitude in our sample of physiological participants might limit the effects of the techniques, while there was room to improve in the work by Castellote‐Caballero et al (Castellote‐Caballero et al., 2013). Second, our experiments were designed to evaluate acute effects (intra‐session), whereas Castellote‐Caballero et al (Castellote‐Caballero et al., 2013) extended the application of the protocol for 3 days along 1 week. Thus, it is conceivable that NM is effective if applied repeatedly over time in subjects with compromised ROM. Some other works involving healthy participant showed that both NM and ST techniques increase ROM intra‐session, but the specific effect of ROM testing maneuver itself was not controlled (Herrintong, 2006; Pietrzak & Vollaard, 2018). It is remarkable that ROM increased in our study along the testing times in all groups (including the ROM‐testing control group, with no interventions); this might be the reason why some studies observed increased ROM during the execution of NT while testing elbow extension (Beneciuk et al., 2009). A similar cumulative effect has recently been described during some protocols of testing muscle force and the level of central drive to the muscle (Madrid et al., 2018).

### 4.2 Effect of the protocol on spinal and muscle excitability

In our work, we observed changes of spinal excitability along the three testing times, a protocol lasting about 90 min. Remarkably, those changes were very small in size and not associated to any intervention because they were present in the Control groups.

First, the latencies of all recorded neurophysiological signals showed small but significant increments. A decrease in muscle temperature, which is reported to increase H and M waves latencies and amplitudes in young healthy subjects (Dewhurst et al., 2005) appears to explain the changes in H and M waves latencies in our work; therefore, changes in muscle temperature might be also the explanation for NT effects in other un‐controlled studies (Castellote‐Caballero et al., 2013; Kumar & Kaur, 2012; Pietrzak & Vollaard, 2018). Our experiments were executed under clinical practice settings, and we did not manipulate muscle temperature; subjects remained with bare legs throughout the protocol (about 90 min). For this reason, limb cooling along the experiment was very small (≈1.2 degrees). Despite such small change in temperature, correlation analysis shown an inverse significant association between the change in latency potentials and limb temperature. Such association was not observed for the increase in H‐waves amplitudes. It is noteworthy saying that the changes in temperature in our work was very small, perhaps for this reason the significant increase in H‐amplitude with much larger reductions in temperature observed previously (Herrintong, 2006) is not questioned by our results. On the other hand, M‐waves amplitudes did not change, but the amplitude of M‐wave for H_50_ was controlled to warrantee similar level of antidromic propagation during reflex testing, and the effect of muscle cooling seems to be of much lower extent (about 50%) for M‐waves that for H‐waves (Dewhurst et al., 2005). We also observed a trend for muscle excitability reduction along the experiment (amplitude of the soleus CMAP), not enough to express changes comparing the different time‐points. This trend might be produced by the prolonged period of absent muscle activity during the protocol (Cupido et al., 1996).

Other works have also reported and effect of the NT techniques on the latencies of H‐reflex. Regrettably, the lack of a proper control group and the fact that in some of them a consistent location of the stimulation electrode cannot be guaranteed, hinders any comparison (Adel, 2011; Kumar et al., 2013; Shaker & Abd El‐Mageed, 2008). This makes possible that the attributed effects of neurodynamics on H‐reflex latency can be explained by some methodological elements not properly controlled.

### 4.3 Limitation of the study

Despite the fact that 60 subjects were enrolled, the groups included were not very large. Subjects were split in groups of 10. This can always have an impact on the power of observations, but the number of subjects in our study were similar to the used in other studies with positive results.

## 5 CONCLUSIONS

One session of neural mobilization techniques does not produce modification of the ROM or spinal excitability in healthy people. However, based on literature, it appears possible a positive aftereffect of NT after multi‐session protocols or single sessions with different application times. This possibility deserves to be studied; however, the use of proper control conditions should be carefully considered, including the control of the aftereffects of ROM testing maneuvers on ROM amplitude.

## AUTHOR CONTRIBUTIONS

**Álvaro Cancela**: conceptualization, data curation, formal analysis, investigation, software, writing—review and editing. **Pablo Arias**: conceptualization, data curation, formal analysis, investigation, methodology, project administration, resources, supervision, validation, writing—original draft. **Beatriz Rodríguez‐Romero**: conceptualization, data curation, investigation, methodology, visualization, writing—review and editing. **Marcelo Chouza‐Insua**: conceptualization, formal analysis, methodology, resources, supervision, writing—review and editing. **Javier Cudeiro**: conceptualization, project administration, supervision, writing—review and editing.

## CONFLICT OF INTEREST STATEMENT

No conflicts of interest, financial or otherwise, are declared by the authors.

## ETHICS STATEMENT

The participants gave their informed consent prior to their inclusion in the study.

## FUNDING INFORMATION

This research did not receive any specific grant from funding agencies in the public, commercial, or not‐for‐profit sectors.
